# Genomic factors limiting the diversity of Saccharomycotina plant pathogens

**DOI:** 10.1093/g3journal/jkaf184

**Published:** 2025-08-12

**Authors:** Sun Lee, Caroline West, Dana A Opulente, Marie-Claire Harrison, John F Wolters, Xing-Xing Shen, Xiaofan Zhou, Marizeth Groenewald, Chris Todd Hittinger, Antonis Rokas, Abigail Leavitt LaBella

**Affiliations:** North Carolina Research Center (NCRC), Department of Bioinformatics and Genomics, The University of North Carolina at Charlotte, Kannapolis, NC 28081, United States; North Carolina Research Center (NCRC), Department of Bioinformatics and Genomics, The University of North Carolina at Charlotte, Kannapolis, NC 28081, United States; Biology Department, Villanova University, Villanova, PA 19085, United States; Department of Biological Sciences, Vanderbilt University, Nashville, TN 37235, United States; Evolutionary Studies Initiative, Vanderbilt University, Nashville, TN 37235, United States; Laboratory of Genetics, Wisconsin Energy Institute, Center for Genomic Science Innovation, J. F. Crow Institute for the Study of Evolution, University of Wisconsin–Madison, Madison, WI 53726, United States; DOE Great Lakes Bioenergy Research Center, University of Wisconsin–Madison, Madison, WI 53726, United States; Centre for Evolutionary and Organismal Biology, Institute of Insect Sciences, Zhejiang University, Hangzhou, Zhejiang 310027, China; Guangdong Province Key Laboratory of Microbial Signals and Disease Control, Integrative Microbiology Research Center, South China Agricultural University, Guangzhou, Guangdong 510642, China; Westerdijk Fungal Biodiversity Institute, Uppsalalaan 8, Utrecht, CT 3584, The Netherlands; Laboratory of Genetics, Wisconsin Energy Institute, Center for Genomic Science Innovation, J. F. Crow Institute for the Study of Evolution, University of Wisconsin–Madison, Madison, WI 53726, United States; DOE Great Lakes Bioenergy Research Center, University of Wisconsin–Madison, Madison, WI 53726, United States; Department of Biological Sciences, Vanderbilt University, Nashville, TN 37235, United States; Evolutionary Studies Initiative, Vanderbilt University, Nashville, TN 37235, United States; North Carolina Research Center (NCRC), Department of Bioinformatics and Genomics, The University of North Carolina at Charlotte, Kannapolis, NC 28081, United States; Center for Computational Intelligence to Predict Health and Environmental Risks (CIPHER), The University of North Carolina at Charlotte, Charlotte, NC 28223, United States

**Keywords:** Saccharomycotina, fungi, phytopathogen, reverse ecology

## Abstract

The Saccharomycotina fungi have evolved to inhabit a vast diversity of habitats over their 400-million-year evolution. There are, however, only a few known fungal pathogens of plants in this subphylum, primarily belonging to the genera *Eremothecium* and *Geotrichum*. We compared the genomes of 12 plant-pathogenic Saccharomycotina strains with 360 plant-associated strains to identify features unique to the phytopathogens. Characterization of the oxylipin synthesis genes, a compound believed to be involved in *Eremothecium* pathogenicity, did not reveal any differences in gene presence within or between the plant-pathogenic and plant-associated strains. A reverse-ecological approach, however, revealed that plant pathogens lack several metabolic enzymes known to assist other phytopathogens in overcoming plant defenses. This includes L-rhamnose metabolism, formamidase, and nitrilase genes. This result suggests that the Saccharomycotina plant pathogens are limited to infecting ripening fruits as they are without the necessary enzymes to degrade common phytohormones and secondary metabolites produced by plants.

## Teaser

Fungi that are pathogenic to plants seriously threaten crops and ecosystems. Some of these plant-pathogenic fungi belong to the subphylum Saccharomycotina. To improve our understanding of plant-pathogenic Saccharomycotina, this work compares the genome content of plant-pathogenic and plant-associated fungi. Lee *et al*. find that plant-pathogenic Saccharomycotina fungi lack genes for enzymes previously shown to be important in the ability of other fungi to infect plants. This work suggests that plant-pathogenic Saccharomycotina are limited to infecting vulnerable plant parts such as fruits.

## Introduction

Across the fungal kingdom, plant pathogens are highly diverse, with a concentration in the phyla Ascomycota and Basidiomycota (Doehlemann et al. 2017). Despite containing over 1,000 species that have evolved over the past 400 million years (Opulente et al. 2024), the subphylum Saccharomycotina, which belongs to the Ascomycota, contains only 1 well-studied plant-pathogenic genus: *Eremothecium.* Recent work has shown, however, that of 1,088 Saccharomycotina strains examined, 33% (360) were isolated directly from living or decaying plants (Harrison et al. 2024; Opulente et al. 2024). It is unknown why phytopathogens are relatively rare in the Saccharomycotina when so many are found in association with plants.

The most well-characterized plant-pathogenic Saccharomycotina species belong to the *Eremothecium* genus of the Saccharomycetales order. These fungi include *Eremothecium gossypii, Eremothecium ashbyi, Eremothecium coryli, Eremothecium sinecaudum*, *Eremothecium cymbalariae,* and *Eremothecium peggii,* which cause rot in a variety of plants, including citrus (Batra 1973; Crous et al. 2021), cotton bolls, coffee, soybean, tomato (Kurtzman et al. 2011), flax (Arnaud 1913), and mustard (Holley et al. 1984). The *Eremothecium* can cause significant crop damage (Batra 1973), yet relatively little is known about the mechanisms of pathogenesis in this group (Perez-Nadales et al. 2014). Oxylipin-covered ascospores in *E. sinecaudum* use a water-driven drilling movement to release spores—a mechanism that could facilitate plant infection (Leeuw et al. 2006). In addition, decreased pathogenicity was observed in *Eremothecium* when oxylipin production was interrupted through exposure to aspirin (Leeuw et al. 2007).

Several other species in the Dipodascales order are known to be pathogenic to plants. *Geotrichum candidum* and *Geotrichum citri-aurantii* commonly cause sour rot in crops, including citrus and tomatoes (Butler et al. 1965, 1988; Wells 1977; Morris 1985; Wild 1987). Similarly, *Geotrichum galactomycetum* has been reported to cause damage to tomatoes and lemons (Butler and Petersen 1972) and *Geotrichum reessii* has been reported as the agent of sour rot in tomatoes (Suwannarach et al. 2016). Other Saccharomycotina exhibit some qualities of plant pathogenesis but do not cause the level of losses to require widespread treatment with fungicides. *Kluyveromyces marxianus* (order Saccharomycetales) was observed to cause onion soft rot (Schroeder et al. 2007). *Botryozyma nematodophila* (order Trigonopsidales), in association with the free-living nematode *Panagrellus zymosiphilus*, has been isolated from the sour rot of grapes, but it is unclear if it is the causative agent of the infection as sour rot is a complex ecological system (Kurtzman et al. 2011; Hall et al. 2018). Recent efforts have provided the genome sequences, functional annotations, and growth characterizations for nearly all known plant-pathogenic species in the subphylum (Opulente et al. 2024) except for *E. ashbyi* and *E. peggii*. This new dataset allows us to identify genomic characteristics unique to Saccharomycotina plant pathogens.

Here, we utilize both a forward and a reverse ecology framework to identify genetic features characteristic of the plant-pathogenic Saccharomycotina. We first identified the genes believed to be involved in oxylipin production in *Eremothecium.* These 3-hydroxy oxylipin synthesis genes were found to be broadly distributed across all the fungi with no notable differences in plant-pathogenic fungi. Next, we conducted a pathway enrichment analysis to identify differences between plant-associated and plant-pathogenic fungi. This revealed that plant-pathogenic Saccharomycotina generally lacked enzymes required for rhamnose and nitrate metabolism. These results were unexpected given previously reported roles for these metabolic pathways in fungal plant pathogens (Bolton and Thomma 2008; Santhanam et al. 2017).

## Materials and methods

### Data source

Genomes, annotation, isolation environment, and phylogeny for 1,154 Saccharomycotina strains were obtained from published work (Opulente et al. 2024). Genera for which no living culture was available or those described after February 2021 were not included in Opulente et al. (2024) and, therefore, not included in our study. Species names (Supplementary Table 1) are accurate as of February 2021 except for the 12 plant-pathogenic strains (Table 1), which have been updated as of February 2025. We defined plant-associated strains as those isolated directly from plants as defined by the Ontology of Yeast Environments (OYE) (Supplementary Table 1; Harrison et al. 2024). Strains pathogenic to plants were defined from the literature and were restricted to Saccharomycotina that were associated with damage to live plants. Fungi that were found associated with rotting plants or fruits were not considered plant-pathogenic. Strains characterized as plant pathogens and their associated references are shown in Table 1.

**Table 1.** Saccharomycotina classified as plant pathogens.

Yeast species	Order	Genome strain	Pathogenicity	Vector
*Geotrichum galactomycetum* (syn. *Galactomyces geotrichum*)	Dipodascales	NRRL Y-17569	Some virulence on tomato and lemon (Butler and Petersen 1972)	Potentially through contaminated equipment as a “machinery mold” (Splittstoesser et al. 1977; Cai and Snyder 2019)
*Geotrichum reessii* (syn. *Galactomyces reessii*)	Dipodascales	NRRL Y-17566	Cause of sour rot in tomato (Suwannarach et al. 2016)	Unknown
*Geotrichum candidum*	Dipodascales	CLIB 918 and CBS 178.71	Cause of sour rot in tomato (Ahmed et al. 2017; Birgen 2017), corn (Mirzaee et al. 2007), carrot (Horita and Hatta 2016), peaches (Yaghmour et al. 2012), strawberry (Ma et al. 2018), nectarine (Yaghmour et al. 2012), kiwi (Cheng et al. 2021) and citrus fruits (Baudoin and Eckert 1982)	Nitidulid beetles and fruit flies (Yaghmour et al. 2012; Ramirez-Camejo et al. 2017) and potentially through contaminated equipment as a “machinery mold” (Cai and Snyder 2019)
*Geotrichum citri-aurantii* (syn. *Galactomyces citri-aurantii*)	Dipodascales	CBS 176.89	Pathogen of citrus fruits, including lemons, oranges, grapefruit, and tangerines (Smith 1917; Butler et al. 1988; Suprapta et al. 1995)	Likely fruit flies and other insects (Hershenhorn et al. 1992)
*Eremothecium coryli*	Saccharomycetales	CBS 5749	Pathogen of cotton bolls (stigmatomycosis) (Esquivel and Medrano 2019), hazelnuts (Hojjati et al. 2023), tomatoes (Miyao et al. 2000), and beans (Kimura et al. 2009; Zecevic et al. 2023)	Sap-sucking pentatomid (Hemiptera) insects, including *Acrosternum hilare* (Glarke and Wilde 1970) and *Nezara viridula* (Esquivel and Medrano 2019)
*Eremothecium cymbalariae*	Saccharomycetales	DBVPG 7215	Pathogen of flax (Arnaud 1913) and hazelnuts (Bobev et al. 2018)	Neotype strain (CBS H-7066) was isolated from plant-pathogenic insect (*Brachynema germari*; Kurtzman et al. 2011)
*Eremothecium gossypii* (syn. *Ashbya gossypii*)	Saccharomycetales	ATCC 10895	Pathogen of cotton bolls (stigmatomycosis) (Ashby and Nowell 1926), coffee, soybean, and other crops (Pridham and Raper 1950)	Plant-feeding insects in the suborder Heteroptera (Dietrich et al. 2013)
*Eremothecium sinecaudum*	Saccharomycetales	ATCC 58844	Pathogen of mustard seed (Holley et al. 1984)	Was isolated from the *Heteroptera* false chinch bug *Nysius ericae* (Burgess et al. 1983; Burgess and Weegar 1986)
*Kluyveromyces marxianus*	Saccharomycetales	DMB1 and NRRL Y-8281	Soft rot pathogen onions (Schroeder et al. 2007)	Spread through infected onions (Schroeder et al. 2007)
*Botryozyma nematodophila*	Trigonopsidales	NRRL Y-17705	Potential role in sour rot of grapes (Smith et al. 1992)	Associated with the nematode *Panagrellus zymosiphilus* and vectorized by Drosophila (Shann 1987a,b)

Based on the literature, we identified 10 species (12 strains total) of yeasts that are likely pathogens. The known plant host and possible vectors are also listed. These fungi primarily infect fruits and are vectorized by insects.

Genome quality metrics and phylogenetic tree were also obtained from previous work (Opulente et al. 2024). Differences in genome metrics were assessed using a phylogenetic ANOVA implemented in the R package phytools (v2.4-4) (Revell 2024). This method automatically conducts post hoc testing for comparison between groups.

### Gene enrichment analysis

We conducted a gene enrichment analysis to identify the pathways and modules enriched or depleted in the plant-pathogenic as compared with plant-associated strains. Kyoto Encyclopedia of Genes and Genomes (KEGG) Orthologs (Kanehisa and Goto 2000) in each ecological group were split into those present in >80% of the genomes and those present in <20% of the genomes (Figshare Data). The R package clusterProfiler v 4.101.1 (Yu et al. 2012) was used to find enriched and depleted genes in each category using all KEGG annotations present in any of the 1,154 genomes as the possible universe. We removed any pathways associated with “Human Diseases” or “Organismal Systems” as they have limited applicability to single-celled fungi. *P*-values were corrected for multiple testing using false discovery rate correction. Pathways that were enriched or depleted in both the plant-associated or plant-pathogenic were filtered from the analysis.

### Gene identification methods

For several genes of interest, we further refined the annotation. We identified reference sequences for these genes using either NCBI protein BLAST (Sayers et al. 2025) results or the genes identified in the previous KEGG annotation (Opulente et al. 2024). The reference proteins (Figshare Data) were used to generate profile hidden markov models (HMMs) using the HMMer v3.3.2 (hmmer.ogr) package. We found amino acid sequences in the genome annotations with HMMERSearch with *E*-values <1 × 10^−50^ and the profile HMM. Finally, we compared these results with the previously identified orthogroups (Opulente et al. 2024). Combining sequence similarity (KEGG and HMMer) and evolutionary information (orthogroups) allowed us to confidently identify the genes within each genome.

We also built gene trees to visually inspect the evolution of the specific genes of interest. Amino acid sequences were aligned using MAFFT v7.273 (Katoh et al. 2002) and gene trees were constructed using IQ-Tree v2.1.2 (Minh et al. 2020), which included the identification of gene models. Trees were visualized in iTOL (Letunic and Bork 2021).

## Results and discussion

### Diversity of plant-pathogenic and plant-associated Saccharomycotina

**Fig. 1.**
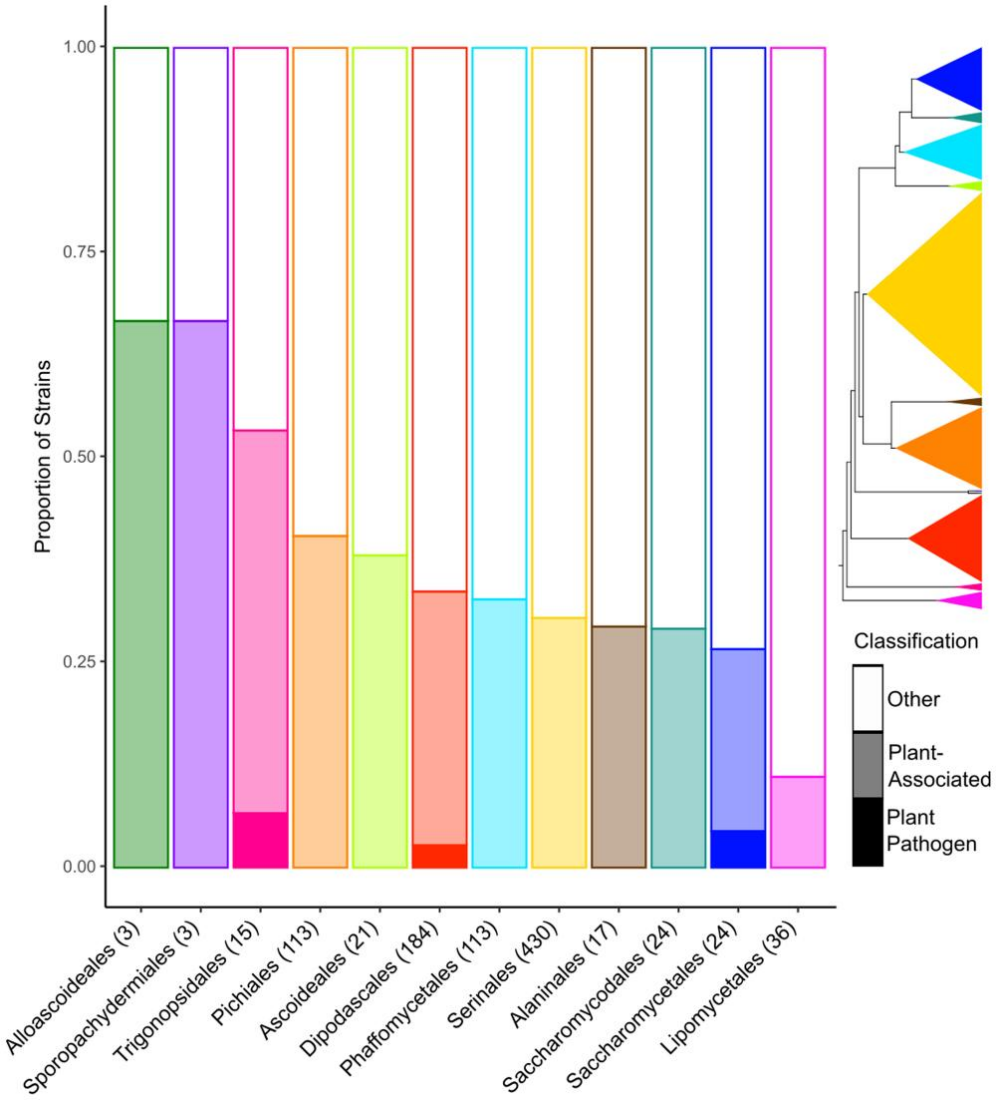
Taxonomic distribution of plant-associated (light color) and plant-pathogenic (dark color) Saccharomycotina. All examined orders have strains that were isolated directly from plants. Conversely, the plant-pathogenic fungi were only found in 3 orders. The total number of strains within each order with known isolation environment information is shown in parentheses. The colors correspond to the orders shown on the phylogeny to the right. The width of the orders is relative to the number of genomes examined.

We divided the Saccharomycotina into 12 plant-pathogenic strains and 360 strains isolated directly from plants based on the yeast isolation environment ontology (Supplementary Table 1; Harrison et al. 2024). We also leveraged the higher-level ecological categorizations from the ontology, which included plant, arthropod, chordate, environmental, and victual (food and drink) associations. All categorizations were made at the strain level. The taxonomic orders of plant-associated strains are shown in Fig. 1. All 12 orders within the Saccharomycotina (Groenewald et al. 2023) had at least 1 plant-associated member, while the plant-pathogenic strains were found in 3 orders: Trigonopsidales, Dipodascales, and Saccharomycetales. At the species level (accounting for 1 species with 2 strains) there were 4 plant-pathogenic Dipodascales (*G. candidum*, *G. galactomycetum*, *G. citri-aurantii*, and *G. reessii*), 1 Trigonopsidales (*B. nematodophila*), and 5 Saccharomycetales (*E. gossypii*, *E. sinecaudum*, *E. coryli*, *E. cymbalariae*, and *K. marxianus*) species. This suggests at least 4 independent origins of plant pathogenesis within the Saccharomycotina. These results highlight the broad taxonomic diversity among the plant-associated and plant-pathogenic Saccharomycotina.

### Genome quality assessment

The genomes analyzed originated from several different sources (Supplementary Table 1). To assess if genome quality between the groups of Saccharomycotina would influence our results, we compared multiple genome statistics between the plant-associated, plant-pathogenic, and all other sampled fungi. We used phylogenetic ANOVAs to compare the following metrics: total length, GC content, N50, Ns per 100 kbp, percent complete BUSCO genes, and percent missing BUSCO genes (Supplementary Table 2). The only significant difference identified was in Ns per 100 kbp between plant pathogens and plant-associated (corrected ad hoc *P*-value = 0.007) or other fungi (corrected ad hoc *P*-value *P* = 0.003). The mean Ns per 100 kbp in the plant pathogens was 807, while the other groups were 59 (plant-associated) and 62 (other). This value is driven by the genome for *Geotrichum candidum* which as 7,825 Ns per 100 kbp, which is the highest value in the entire dataset. Despite the high value for this metric, *Geotrichum candidum* has 93.96% complete BUSCO and an N50 of 1,159,651. This analysis indicates that there are no significant differences in genome quality or annotation between the plant-associated and plant-pathogenic fungi examined.

### 3-Hydroxy oxylipin synthesis

Oxylipins are important secondary metabolites frequently produced by fungi (Sebolai et al. 2012). In *Eremothecium*, 3-hydroxy oxylipins have a well-characterized role in ascospore release (Smith et al. 2000; Kock et al. 2003) and the sexual cycle (Leeuw et al. 2007). They are also believed to be involved in pathogenesis, potentially by facilitating invasion into plant tissue (Leeuw et al. 2006). The 3-hydroxy oxylipins are believed to be generated through incomplete β-oxidation of fatty acids in the Saccharomycotina (Sebolai et al. 2012). The enzymes that conduct β-oxidation are an acetyl-CoA dehydrogenase and an enoyl-CoA-hydratase (Sebolai et al. 2012). In *Saccharomyces cerevisiae*, the acetyl-CoA dehydrogenase is Pox1 (alias Fox1), and the enoyl-CoA-hydratase is Fox2. Most fungi conduct β-oxidation in the peroxisomes, as opposed to in the mitochondria (Poirier et al. 2006). Emerging evidence, however, suggests that some Saccharomycotina, such as *Clavispora lusitaniae* (syn. *Candida lusitaniae*), have a functional mitochondrial β-oxidation pathway (Gabriel et al. 2014). The mitochondrial and peroxisomal β-oxidation pathways both utilize the enzyme Fox2, suggesting enzymatic overlap between the peroxisomal and mitochondrial β-oxidation pathways. We investigated the presence of the acetyl-CoA dehydrogenase and enoyl-CoA-hydratase enzymes across the plant-associated and plant-pathogenic strains.

The acetyl-CoA dehydrogenase converts fatty acyl-CoA to trans-2-enoyl-CoA (Hiltunen et al. 2003). The *S. cerevisiae* acetyl-CoA dehydrogenase Pox1 belongs to the KEGG Orthogroup (KO) K00232. We identified 2,213 genes in the KO K00232 across all the strains (Figshare Data). Of the 2,213 genes, we identified 23 putative horizontal gene transfer events of a gene from outside the Saccharomycotina subphylum based on the Pox1 gene tree structure and blast results (Figshare Data). The gene was not detected in 30 strains, including all 24 Saccharomycodales species in this study. The gene tree structure suggests a shared ancestry for the remaining 2,190 sequences with multiple duplication events. There were likely 2 duplication events in order Serinales, where 54% of species had 3 paralogs and 38% had 2 paralogs. Similarly, 37% of Dipodascales species had 2 paralogs of this gene, and 92% of Phaffomycetales had 2 or more paralogs. All plant-pathogenic Saccharomycotina, including the *Eremothecium,* had at least 1 acetyl-CoA dehydrogenase gene.

The enoyl-CoA-hydratase converts trans-2-enoyl-CoA to 3-ketoacyl-CoA and is known as Fox2 in *S. cerevisiae*, which maps to the KO K14729 (Hiltunen et al. 2003). We identified 1,159 genes in the KO (Figshare Data). Like the acetyl-CoA dehydrogenase, the Fox2 gene encoding the enoyl-CoA-hydratase was absent in 36 strains, including all the Saccharomycodales species sampled here. The gene sequences generally fell within their orders except for the Alaninales, which were nested within the Pichiales instead of as a sister clade. All plant-pathogenic strains, including the *Eremothecium*, had at least 1 enoyl-CoA-hydratase gene.

Based on gene presence and absence data, the oxylipin synthesis genes are not clearly associated with pathogenesis despite their role in *Eremothecium* development and aspirin sensitivity. Additional experiments and analysis will be needed to complete our understanding of how oxylipin synthesis enzymes contribute to Saccharomycotina plant pathogenesis.

### Identification of pathways that differ between plant-associated and plant-pathogenic strains

To identify pathways that distinguish plant-associated from plant-pathogenic strains, we categorized KOs as present in 80% or more of the strains from each group and present in less than 20% of the strains from each group. We then performed a KEGG pathway enrichment analysis on the KEGGs present (>80%) and absent (<20%) in the plant-associated and plant-pathogenic strains (Supplementary Table 3). These data were then filtered to identify pathways enriched in only 1 group. There were 12 pathways uniquely enriched in these categories (Table 2). Most of these results (10 pathways) were unique to the plant-pathogenic strains. The 2 pathways with the lowest *P*-values for an association with plant pathogens were “fructose and mannose metabolism” and “nitrogen metabolism.”

**Table 2.** KEGG pathway enrichment analysis of genes present or absent in plant-associated and plant-pathogenic Saccharomycotina.

Gene category	Ecological category	KEGG pathway	Description	Adjusted *P*-value
Absent	Plant-associated	ko00280	Valine, leucine and isoleucine degradation	0.002
**Absent**	**Pathogenic**	**ko00051**	**Fructose and mannose metabolism**	**0**.**002**
**Absent**	**Pathogenic**	**ko00910**	**Nitrogen metabolism**	**0**.**003**
Present	Plant-associated	ko00563	Glycosylphosphatidylinositol-anchor biosynthesis	0.009
Absent	Pathogenic	ko00350	Tyrosine metabolism	0.020
Present	Pathogenic	ko04110	Cell cycle	0.020
Absent	Pathogenic	ko00052	Galactose metabolism	0.022
Present	Pathogenic	ko00290	Valine, leucine, and isoleucine biosynthesis	0.024
Present	Pathogenic	ko04068	FoxO signaling pathway	0.033
Absent	Pathogenic	ko02024	Quorum sensing	0.034
Absent	Pathogenic	ko02020	Two-component system	0.035
Absent	Pathogenic	ko01110	Biosynthesis of secondary metabolites	0.042

The most significant pathways identified in plant-pathogenic fungi that were not identified in plant-associated yeasts were fructose and mannose metabolism and nitrogen metabolism (in bold).

The “fructose and mannose metabolism” pathway result was driven by the complete lack of rhamnose metabolism genes in the plant-pathogenic strains. Four enzymes convert L-rhamnose into L-lactaldehyde, and all of them are absent in the plant-pathogenic strains (Supplementary Table 4). The 4 enzymes are L-rhamnonate dehydratase (K12661), L-rhamnose 1-dehydrogenase (K18337), 2-keto-3-deoxy-L-rhamnonate aldolase (K18339), and L-rhamnono-1,4-lactonase (K18338). These enzymes are found in 34%, 34%, 47%, and 33% of plant-associated strains, respectively. Fisher's exact test also showed significant differences in enzyme counts between plant-associated and plant-pathogenic fungi with *P*-values of 0.0114 (K18338), 0.0007 (K18339), 0.0107 (K18337), and 0.0105 (K12661) (Supplementary Table 5). Overall, 31% (110/360) of the plant-associated strains had a complete L-rhamnose metabolism pathway compared with none of the plant-pathogenic strains. Unsurprisingly, none of the plant-pathogenic strains can grow on L-rhamnose when tested in the laboratory (Kurtzman et al. 2011; Opulente et al. 2024), while 16% (43/265) of the plant-associated strains with growth data were able to grow on L-rhamnose (Supplementary Table 6). Across all strains measured, 15% of strains could grow on L-rhamnose. The lack of L-rhamnose metabolism genes is somewhat surprising, given that L-rhamnose is widely found in plants (Jiang et al. 2021). In plants, L-rhamnose is found in the cell wall and is used to make specialized metabolites, including glycoalkaloids (Jiang et al. 2021). In tomatoes and potatoes, L-rhamnose is needed to produce steroidal glycoalkaloids, which are used to defend against microbial and insect pests (McCue et al. 2007).

### Underrepresentation of nitrogen metabolism genes in plant-pathogenic strains

**Fig. 2.**
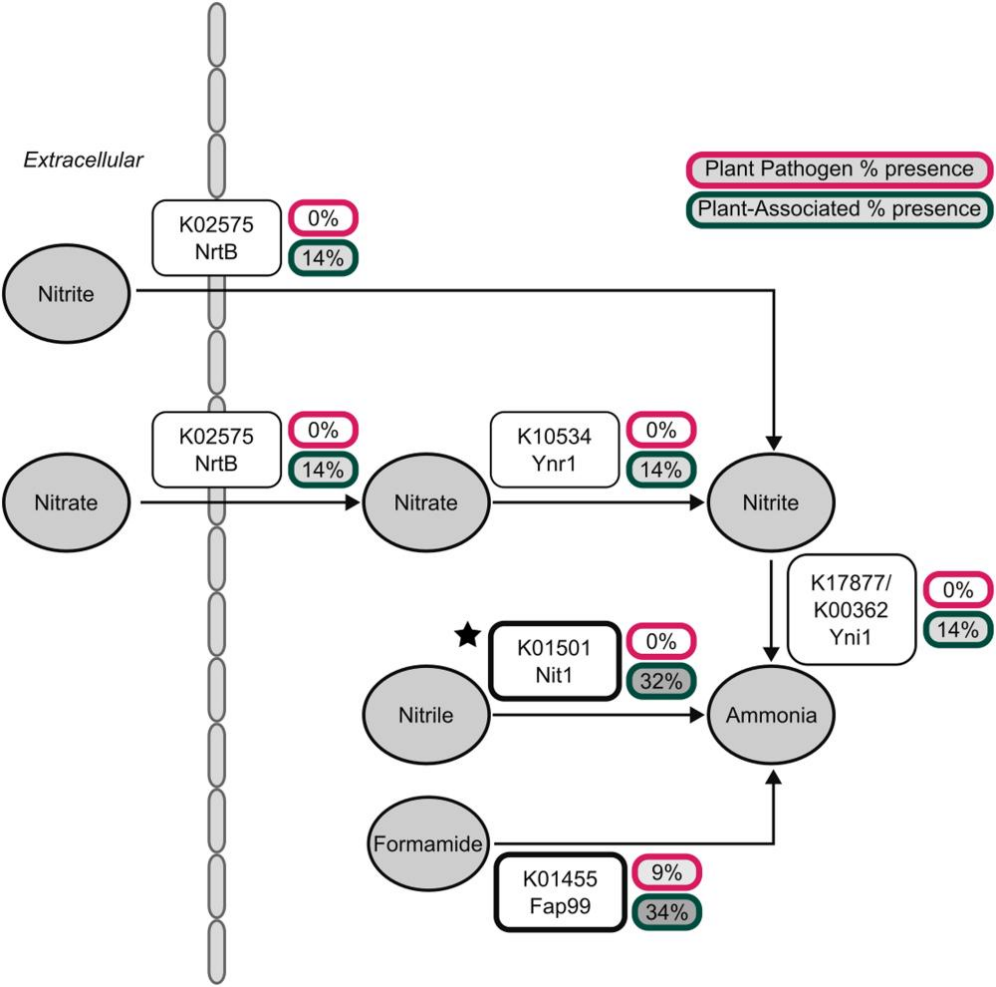
Presence of nitrogen metabolism genes identified in the pathway enrichment analysis. The plant-pathogenic strains almost entirely lack the necessary enzymes to metabolize nitrate, nitrile, or formamide into ammonia. This suggests that these strains cannot use nitrate or nitrite as their sole nitrogen source. Fisher's exact test showed a significant difference (star) in K01501 between plant-associated and plant-pathogenic fungi (*P*-value = 0.021).

The nitrogen metabolism pathway was underrepresented in the plant-pathogenic but not the plant-associated fungi. Six KOs in the nitrogen metabolism pathway were completely absent or present in only 1 plant-pathogenic strain but present in 14% or more of plant-associated strains (Supplementary Table 7, Fig. 2). Each of these enzymes is involved in the processing of nitrogen-containing compounds into ammonia.

The nitrite reductase gene *YNI1*, previously characterized in *Ogataea polymorpha* (Siverio 2002), is associated with 3 KOs (K17877, K00362, and K00363). This gene is found in a gene cluster with the nitrate reductase gene *YNR1* (K10534) (Siverio 2002), which was also identified in this analysis. The other genes in this cluster are the nitrate transporter *YNT1* (K02575) and the transcription factor encoding genes *YNA1* and *YNA2* (no KEGG association). We identified strains with putative nitrate assimilation gene clusters by identifying genomes with *YNI1*, *YNR1*, and *YNT1* KOs within a 10-gene window (Supplementary Table 8, Figshare Data). We found that Saccharomycotina strains isolated from victuals (food or drink) had the highest proportion of genomes with the nitrate assimilation cluster (15 out of 92 genomes) and that Alaninales had the highest proportion (12 out of 17 genomes). In the 12 plant pathogen genomes, we identified no nitrate assimilation gene clusters. We also did not find a strong association between this cluster and the other environmental categories—the proportion of strains with this cluster ranges from 8% to 16% in arthropod, chordate, environmental, plant, and victuals categories. Given that this cluster is generally rare, we do not suspect that this cluster's absence contributes to plant-pathogenicity.

We also identified a lack of the nitrate/nitrite transporter (K02575), encoded by *NRTB* in *O. polymorpha*, in the plant-pathogenic Saccharomycotina. Across all the strains, the transporter was found in 159 genomes, including all genomes predicted to have the nitrate/nitrite assimilation cluster (125 genomes). The proportion of this gene is similar across environments, ranging from 11% in chordate-associated (8 of 74) to 17% in victuals-associated (16 of 92; Supplementary Table 9).

The KO K01455 was found in 1 plant-pathogenic strain (*B. nematodophila*) and 34% (122/238) plant-associated strains. This KO is annotated as a formamidase and is associated with the gene *FAP99* in the human-pathogenic species *Candida albicans.* In general, this enzyme hydrolyzes formamide into formic acid but has not been extensively studied in Saccharomycotina. It is known, however, that formamide is produced during cyanide degradation and that formamidases further metabolize formamide as a carbon or nitrogen source (Malmir et al. 2022). In the filamentous phytopathogenic fungus *Verticillium dahliae*, the regulation of the formamidase gene is critical to pathogenicity (Xiao et al. 2025). In this system, overexpression of formamidase, induced by deletion of a negative regulated, led to decreased virulence. The lack of formamidase in the majority of plant-pathogenic strains suggests they cannot use formamide as a nitrogen or carbon source and are unlikely to be able to metabolize cyanide produced by plants as a defense.

Finally, we analyzed the nitrilase (K01501), which was observed to be absent in the plant-pathogenic strains. This gene is characterized as *NIT1* in *S. cerevisiae.* The nitrilase KEGG annotation overlapped much less with the nitrate/nitrite assimilation cluster. Of the 369 genomes with a K01501 annotation, only 59 had the cluster. Across the orders, the K01501 annotation was found in all orders except for Alloascoideales, which has genomes available from 3 species (Supplementary Table 10). The median presence was 33%, ranging from 13% in the Trigonopsidales (2 of 15) to 96% in the Saccharomycodales (23 of 24). The K01501 annotation was also widely distributed across strains isolated from different environments. It was found in approximately one-third of strains from all environments. The proportion of plant-associated strains with a nitrilase gene was 31% (114/360). This led to the hypothesis that the loss of nitrilases is a distinguishing feature of plant pathogens compared with plant-associated strains. Interestingly, plant-pathogenic and plant growth-promoting microorganisms have been shown to produce nitrilases (Barclay et al. 1998). Loss of nitrilases and other nitrilase superfamily enzymes, like cyanide hydratases and cyanide dihydratases, could result in a loss of cyanide and nitrile detoxification. For example, the rhizobacterium *Pseudomonas fluorescens* SBW25 produces a nitrilase, which allows it to tolerate a toxic level of nitriles produced by plants (Howden and Preston 2009). Similarly, in the filamentous plant pathogen *Fusarium solani,* a cyanide hydratase allows this fungus to tolerate toxic levels of cyanide (Barclay et al. 1998). None of the Saccharomycotina, however, have enzymes mapped to cyanide hydratase (K10675) or cyanide dihydratase (K18282).

### Diversity of nitrilase genes in Saccharomycotina

In addition to *NIT1*, at least 2 other nitrilases are present in Saccharomycotina and are known as *NIT2* and *NIT3*. These enzymes belong to the nitrilase superfamily (Pace and Brenner 2001).To capture the full diversity of nitrilases in the subphylum, we conducted a thorough search of the genome annotations. We identified 3,963 putative nitrilase genes in an HMMer search using the KEGG annotated genes as the reference (Figshare Data). We then assigned these hits to previously calculated orthogroups (Opulente et al. 2024). Five distinct nitrilase orthogroups were identified: OG0003182 (748), OG0003106 (1143), OG000784 (1335), OG0004284 (550), and OG0004905 (186). The orthogroups were characterized as follows by comparing them to the KEGG Data: OG0004284 to *NIT1* and K01501, OG0003106 to *NIT2* and K11206, and OG000784 to *NIT3* and K13566. The orthogroup OG0004905 was also associated with K13566. OG0003182 corresponded to *NTA1* in *C. albicans* and K14663. The orthogroups OG0004284 (*NIT1*), OG0003106 (*NIT2*), and OG0000784 (*NIT3*) had much higher similarity to the nitrilase reference sequences (median *e*-values/bit scores of 3.95 × 10^−83^/277.3, 6.8 × 10^−81^/270.0, and 9.4 × 10^−92^/305.7, respectively) relative to OG0004905 and OG0003182 (median *e*-values/bit scores of 1.15 × 10^−53^/180.8 and 8 × 10^−59^/197.7, respectively). The orthogroup OG0003182 corresponds to the KO K14663 and is NTA1 in *S. cerevisiae*. NTA1 is involved in the Arg/N-end rule pathway of protein degradation (Kim et al. 2016). Given the relatively higher *e*-values, bit scores, and associated KOs of OG0004905 and OG0003182, these were unlikely to be nitrilases and are not discussed further.

The pathway enrichment analysis identified the *NIT1* genes belonging to OG0004284. The comprehensive HMMER search identified an additional 85 *NIT1* instances in 73 species and did not include 9 previously identified instances in 4 species. However, the proportions of this gene in plant-pathogenic (0%: 0/12) and plant-associated (33% 120/360) species were not significantly different from the KO-based analysis reported above (Supplementary Table 11).

The gene *NIT2* encodes an amidase of deaminated glutathione involved in the metabolism and maintenance of glutathione (Peracchi et al. 2017), which is an antioxidant involved in plant development and stress responses (Bela et al. 2015). This enzyme was present in 88% of plant-associated (318/360) and 36% of plant-pathogenic strains (5/12; Supplementary Table 11). The *NIT2* orthogroup was found in *K. marxianus*, *G. citri-aurantii*, *G. reessii, G. galactomycetum*, and 1 of 2 stains of *G. candidum* (CLIB 918 and not CBS 178.71). Glutathione plays a critical role in plant signaling and plant defense against pathogens (Dubreuil-Maurizi and Poinssot 2012). Deficiencies in plant glutathione synthesis enhance the susceptibility of *Arabidopsis thaliana* to fungal pathogens such as *Botrytis cinerea* (Ferrari et al. 2003). Similar to NIT1, many of the plant pathogens, including the 4 *Eremothecium* species, lost the ability to process deaminated glutathione.

The gene *NIT3* (OG0000784) encodes an omega-amidase and was found in 73% (9/12) of the plant-pathogenic strains and 90% (323/360) of the plant-associated strains (Supplementary Table 11). NIT3 may play a role in biofilm formation as the protein was identified in *C. albicans* biofilm extracts (Martinez et al. 2016) and secretomes (Vaz et al. 2021). These prior findings could indicate that Nit3 functions extracellularly, but this localization has not been demonstrated beyond model fungi.

It is counterintuitive that the loss of formamidase and nitrilase (*NIT1* and *NIT2*) genes was associated with plant-pathogenic Saccharomycotina. Formamidase and nitrilase genes have been shown to be involved in nitrogen assimilation and defense against plant compounds. Studies in other plant-pathogenic fungi suggest that nitrogen limitation occurs early in infection (Bolton and Thomma 2008) and that nitrogen utilization is key for virulent infection (Horst et al. 2012). Nitrilase superfamily enzymes in plant-associated bacteria may play a nitrogen assimilation role (Howden and Preston 2009). Interestingly, plant-pathogenic strains rarely have the ability to assimilate nitrates in growth experiments (Kurtzman et al. 2011; Opulente et al. 2024). We found no plant-pathogenic strains that could assimilate nitrate, while 14% (48/337) of the plant-associated strains had the ability (Supplementary Table 6). These results suggest that paradigms developed around nitrogen assimilation in other Ascomycota plant-pathogenic fungi are unlikely to be applicable in Saccharomycotina.

Plants also use nitrile-containing secondary metabolites as defense mechanisms. For example, cyanogenic glycosides, a type of α-hydroxynitrile, defend against insect herbivores (Gleadow and Moller 2014). Plants also use phenylacetonitrile for defense (Yactayo-Chang et al. 2020). Insects ingest these nitriles and break them down into cyanide, which kills them (Martinez and Diaz 2024). Fungi are known to utilize nitrilase superfamily enzymes to degrade cyanide; this includes species such as *Neurospora crassa*, *Aspergillus nidulans, Fusarium graminearum* (Basile et al. 2008), and *Gloeocercospora sorghi* (Wang et al. 1992). The Saccharomycotina plant-pathogenic strains lack nitrilase enzymes to defend against these compounds.

### Conclusions

Our analysis of plant-pathogenic Saccharomycotina fungi revealed the absence of specific metabolic capabilities in this group. This study, however, is limited by several factors. First, the ecology of each of the more than 1,000 fungal species in this dataset is not well established. Our dataset may contain uncharacterized or opportunistic plant pathogens. In addition, our study was limited to examining gene presence and absence and, therefore, does not account for regulatory changes that contribute to plant pathogenesis. Despite these limitations, the complete loss of the pathways examined in plant-pathogenic Saccharomycotina warrants examination.

One hypothesis for the loss of these pathways is that the vectors have shaped Saccharomycotina plant pathogen evolution. This is the case in the well-studied yeast–cactus–*Drosophila* system, where the 3 organisms exhibit coadaptation (Starmer and Fogleman 1986). The *Eremothecium* are known or theorized to be spread by insects that pierce the fruit and facilitate infection (Table 1). In addition, *G. candidum*, *G. citri-aurantii*, and *B. nematodophila* are likely spread by insects such as fruit flies. Fruit flies can puncture fruit surfaces to deposit larvae, which suggests that they can vectorize plant fungal pathogens. For example, the invasive *Drosophila suzukii* has been associated with the spread of fruit rot pathogens such as *B. cinerea* in strawberries (Lewis et al. 2019). In these cases, the Saccharomycotina rely on the vector to bypass the physical barriers outside of fruits. It is also possible that vectors could produce enzymes that compensate for those lost in the Saccharomycotina. This reliance on vectors for invasion of plant tissue may have limited the evolution of the Saccharomycotina plant pathogens.

Similarly, the Saccharomycotina plant pathogens may have evolved to avoid cyanogenic glycosides. Cotton, a major host for *E. gossypii*, produces secondary metabolites as a defense mechanism, such as terpenes, but is not known to produce cyanogenic glycosides (Stipanovic et al. 2010). Similarly, *G. candidum* causes sour rot in citrus plants, which are also not known to produce cyanogenic glycosides (Ali et al. 2014). This pattern suggests that the presence of cyanogenic glycosides and cyanide, even in small amounts, may prevent the Saccharomycotina plant pathogens from colonizing plant tissue. This is also consistent with the observation that Saccharomycotina pathogens target fruits as opposed to other plant parts like leaves, stems, or roots. Fruit defenses are highly complex and vary during the transition from immature to mature and dispersed fruits (Whitehead et al. 2022). During ripening, fruits undergo morphological and biochemical changes, such as cell wall degradation and changes in phytohormone (including aspirin or salicylic acid) production, which can result in increased susceptibility to fungal infection (Alken and Fortes 2015). For example, immature strawberries contain sufficient proanthocyanidins to prevent *B. cinerea* infection, but as they ripen, the activity of the proanthocyanidins decreases, allowing the fungus to infect the fruit (Jersch et al. 1989). It is also possible that Saccharomycotina plant pathogens are restricted to fruits with low nitrate content. Overall, Saccharomycotina appear to be limited by their vectors and genetic composition to infecting ripening fruits.

## Supplementary Material

jkaf184_Supplementary_Data

## Data Availability

All supplemental data not included in the supplementary tables are hosted on a FigShare repository: https://doi.org/10.6084/m9.figshare.c.7691546. Supplemental material available at G3 online.
