# Supplementary material for: Genomic factors limiting the diversity of Saccharomycotina plant pathogens
Source: G3 (Bethesda). 2025 Aug 12;15(10):jkaf184. doi: 10.1093/g3journal/jkaf184 (PMC12506670; doi:10.1093/g3journal/jkaf184)
Supplement: jkaf184_Supplementary_Data [file jkaf184_supplementary_data.zip › Supplemental_Material_Legends_G3-2025-406050.docx]

**Supplemental Data**

Supplementary Table 1: Strain designation, genome information and taxonomic order for all 1,154 strains in the dataset. This table also includes the environment from which the strain was isolated and the categorization of that environment.

Supplementary Table 2: Statistical analysis of genome quality metrics between plant-associated and plant pathogenic Saccharomycotina. Tests include T-test and Phylogenetic ANOVA with post-hoc testing.

Supplementary Table 3: KEGG pathway enrichment results. The unfiltered results for the KEGG pathway enrichment analysis, including all pathways enriched in both plant-associated and plant-pathogenic strains.

Supplementary Table 4: Presence and absence of rhamnose metabolism genes in plant-associated and plant-pathogenic strains. The strain count and strain percents are shown.

Supplementary Table 5: Fisher’s exact test of KEGG gene presence between plant-associated and plant pathogenic Saccharomycotina.

Supplementary Table 6: Binary growth of strains on rhamnose, nitrate, and nitrite with associated citations

Supplementary Table 7: Presence and absence of nitrilase genes in the plant-associated and plant-pathogenic strains. This includes the raw strain count and percentages.

Supplementary Table 8: Presence of one or more nitrate reductase clusters in the Saccharomycotina.

Supplementary Table 9: Strains with the nitrate/nitrite transporter (K02575), encoded by *NRTB* in *O. polymorpha.*

Supplementary Table 10: Presence and absence of the nitrilase (K01501) gene across the strains.

Supplementary Table 11: Presence of characterized orthogroups in the plant-associated and plant-pathogenic fungi.
